# Plasma lipid profiles and risk of cardiovascular disease in occupational lead exposure in Abeokuta, Nigeria

**DOI:** 10.1186/1476-511X-4-19

**Published:** 2005-09-28

**Authors:** Oladipo Ademuyiwa, Regina Ngozi Ugbaja, Florence Idumebor, Olugbenga Adebawo

**Affiliations:** 1 Department of Biochemistry, University of Agriculture, Abeokuta, Nigeria; 2 Department of Biochemistry, Olabisi Onabanjo University, Ikenne, Nigeria

## Abstract

In order to investigate the effects of lead exposure on risk of cardiovascular disease during occupational exposure to this metal, plasma cholesterol and its fractions as high-density liporotein (HDL), low-density liporotein (LDL) and triglyceride were determined in various artisans in Abeokuta, Nigeria who have been shown to be occupationally exposed to lead and these were related to blood lead levels. Increased risk of cardiovascular disease was observed in the artisans. Total cholesterol in the artisans was between 1.5 and 2.0 times higher in the artisans than that present in controls while LDL cholesterol was between 1.6 and 2.4 times higher in the artisans when compared with control subjects [p < 0.001]. HDL cholesterol and triglyceride levels were not affected [p > 0.05]. A significant positive correlation was observed between blood lead and total cholesterol on one hand [r = 0.372; p = 3.0 × 10^-5^] and blood lead and LDL cholesterol on the other hand [r = 0.283; p = 0.001]. LDL/HDL cholesterol ratio was also higher in the artisans when compared with control. Blood pressure (systolic and diastolic) and other anthropometric parameters were not significantly different between the artisans and the control subjects [p > 0.05]. Results suggest that lead exposure increases cholesterol synthesis and transport to peripheral tissues whereas reverse cholesterol transport to the liver is not affected.

## Introduction

Studies in both humans and animals indicate that lipid metabolism is altered in chronic lead exposure [1-6]. The pathophysiological mechanisms involved in this lead-induced alterations are not completely understood. Lead has been shown to accelerate lipid oxidation in the presence of hemoglobin or Fe^2+ ^[7,8]. Lead was also shown to enhance Fe^2+^-initiated lipid oxidation in liposomes, erythrocytes, microsomal fractions and rat brain homogenates [1,7,9]. Altered fatty acid composition of erythrocyte membranes has also been demonstrated in chronic lead exposure [5,10,11]. It has also been demonstrated that lead exposure affects levels of galactolipid metabolic enzymes in the developing rat brain resulting in myelin defects [3]. All these observations are largely more suggestive than conclusive about lead-induced alterations in lipid metabolism.

Lead poisoning is presently becoming the most common disease of environmental origin and is increasing very rapidly in developing countries [12-16]. While lead exposure studies is a well-trodden subject in economically advanced countries [17-22], few published reports exist about lead poisoning and its associated effects in developing countries like Nigeria [12-14,16].

During the past years, numerous reports have appeared in the literature indicating that abnormal blood lipid levels like total cholesterol and other lipids and lipoproteins predispose individuals to atherosclerosis and cardiovascular diseases [23-27]. We have previously reported on chronic lead poisoning and its associated biochemical effects in auto-mechanics, petrol station attendants and some other artisans in Abeokuta, Nigeria [12-14,16]. To the best of our knowledge, data regarding the distribution of blood lipids and the risk of cardiovascular disease associated with this distribution in those who are occupationally exposed to lead in Nigeria, are lacking. In order to gain an insight into lead exposure and its effects on lipid profiles, we investigated the distribution of blood lipids in various artisans in Abeokuta, Nigeria, who have been shown to be occupationally exposed to lead.

## Subjects and methods

### Study population

A total of 110 male subjects participated in the study. Control subjects were made up of staff and students of University of Agriculture, Abeokuta, Nigeria, while lead-exposed subjects comprised of different artisans located in two mechanic workshops in the southern and northern parts of the city of Abeokuta, Nigeria. It is typical of mechanic workshops in Nigeria to find other groups of artisans located in these workshops in addition to the auto-mechanics, thereby complementing each other's services. In these workshops, there is always a preponderance of auto-mechanics. After explaining the objectives and the requirements of the study to them, a total of 92 artisans, including 50 auto-mechanics, consented to participate in the study. Seven male petrol station attendants from a petrol station in the southern part of the city were also included among the occupationally exposed subjects. Table 1 summarises the study population.

**Table 1:** Study population

Subject	Number
Control	11
Auto-mechanics	50
Auto-electricians	8
Battery chargers	2
Drivers	2
Painters (Vehicle)	7
Panel beaters	15
Petrol station attendants	7
Upholsterers	4
Spare parts/Oil sellers	2
Welders	2

### Anthropometric measurements and body composition

The weight of each subject was measured to the nearest 0.1 kg with a battery operated scale while the subjects were dressed in their underwear and height was measured to the nearest centimeter with the aid of a portable stadiometer. From these data, Body Mass Index (BMI), Body Surface Area (BSA), Body Fat Mass Index (BFMI), Fat Free Mass Index (FFMI),), Lean Body Mass (LBM), Body Fat Mass (BFM), Total Body Water (TBW), Intracellular Fluid (IF) and Extracellular Fluid (EF), were calculated [28-30,35]. Blood pressure was measured two times on the left arm in each subject in a supine position with a traditional sphygmomanometer (Cacosson Products, England) [31]. Each measurement was spaced twenty minutes apart and was usually performed before collection of blood samples. The average of the two measurements was used for all analyses.

### Blood analyses

Venous blood samples were obtained between 8.00 a.m and 10.00 a.m from the subjects after an overnight fast. Aliquots of blood samples were separated for lead analysis and the remaining blood samples were centrifuged to separate plasma and red blood cells. Plasma concentrations of total cholesterol, LDL-cholesterol and triglycerides were determined with commercial kits (Randox Laboratories, Crumlin, England). HDL-cholesterol was determined in plasma with the same commercial kits for total cholesterol after very low density lipoproteins (VLDL) and low density lipoproteins (LDL) were precipitated with heparin-MnCl_2 _solution [32]. Blood lead analysis was performed using atomic absorption spectrophotometry. Details of this have been given elsewhere [13,14].

### Statistical protocol

Results are expressed as mean ± S.D. One-way analysis of variance (ANOVA) followed by the least significant difference (LSD) test were used to analyse the results with p < 0.05 considered significant [33]. The relationships between blood lead levels and plasma lipids and the anthropometric parameters were also analysed using Pearson correlations [33].

## Results

### Demographic and anthropometric characteristics

The demographic and anthropometric characteristics of the subjects are depicted in Table 2. Their ages ranged between 18 and 66 years. The years of experience on the job for each group also shows a wide variation ranging from 0.7 to 53 in the auto-mechanics and 1.5 to 5 in the petrol station attendants. With the exception of the drivers and spare parts dealers, majority of the artisans spend an average of 11 hours per day in their workshops. The petrol station attendants on the other hand, spend an average of 14.3 hours with their nozzle.

**Table 2:** Some demographic and anthropometric characteristics of the subjects. Values are mean ± S.D.

	Subjects										
Parameters	Control	Auto-mechanics	Auto-electricians	Battery chargers	Drivers	Painters (Vehicle)	Panel beaters	Petrol station attendants	Upholsterers	Spare parts/Oil sellers	Welders

Age (years)	26.00 ± 6.65	28.02 ± 10.72	32.00 ± 10.17	27.00 ± 7.07	39.50 ± 12.02	34.00 ± 19.56	29.53 ± 13.03	28.71 ± 3.99	29.25 ± 8.96	30.50 ± 0.70	37.50 ± 3.54
Job experience (years)	-	11.64 ± 10.92	13.25 ± 11.18	7.50 ± 0.71	4.00 ± 2.83	15.71 ± 19.96	12.47 ± 10.45	3.57 ± 1.54	12.38 ± 11.46	6.50 ± 2.12	25.50 ± 0.71
Hours spent/day in workshop	8.64 ± 0.92	11.62 ± 0.78	11.25 ± 1.16	11.50 ± 0.71	10.00 ± 0.00	11.14 ± 0.69	11.20 ± 0.68	14.29 ± 3.25	11.25 ± 1.50	10.50 ± 2.12	11.50 ± 0.71
Height (m)	1.69 ± 0.05a	1.67 ± 0.13a	1.72 ± 0.05a	1.70 ± 0.02a	1.68 ± 0.04a	1.73 ± 0.08a	1.72 ± 0.03a	1.71 ± 0.08a	1.69 ± 0.07a	1.68 ± 0.02a	1.73 ± 0.04a
Weight (kg)	60.64 ± 8.10a	62.44 ± 11.54a	62.38 ± 7.89a	57.50 ± 0.71a	67.00 ± 11.31a	54.33 ± 2.52a	63.71 ± 10.16a	68.00 ± 7.26a	58.25 ± 11.62a	58.00 ± 4.24a	67.50 ± 14.85a
BMI (kg/m^2^)	21.34 ± 3.07a	21.78 ± 3.76a	20.88 ± 2.06a	20.02 ± 0.25a	23.66 ± 2.81b	18.15 ± 1.72a	21.71 ± 3.73a	23.49 ± 3.39b	20.62 ± 4.34a	20.66 ± 0.99a	24.84 ± 0.90b
BSA (m^2^)	1.69 ± 0.11a	1.71 ± 0.14a	1.72 ± 0.10a	1.66 ± 0.03a	1.76 ± 0.16a	1.65 ± 0.08a	1.78 ± 0.12a	1.79 ± 0.10a	1.64 ± 0.20a	1.41 ± 0.29b	1.88 ± 0.09c
BFMI (kg/m^2^)	3.81 ± 1.45a	4.08 ± 2.29a	3.24 ± 1.19a	3.29 ± 0.72a	5.17 ± 1.92a	1.81 ± 0.91a	8.57 ± 4.39c	6.04 ± 2.30b	10.28 ± 8.24d	3.24 ± 0.60a	6.35 ± 0.21b
FFMI (kg/m^2^)	17.53 ± 1.63a	17.44 ± 3.64a	17.48 ± 0.84a	16.73 ± 0.47a	18.50 ± 0.91a	16.35 ± 0.81a	15.88 ± 3.75a	17.44 ± 1.08a	10.34 ± 12.45b	17.43 ± 0.40a	18.46 ± 1.08a
LBM (kg)	49.88 ± 4.66a	50.67 ± 5.78a	51.52 ± 4.72a	47.64 ± 2.03a	53.21 ± 6.61a	47.13 ± 2.37a	52.08 ± 4.92a	50.43 ± 3.14a	47.72 ± 8.42a	48.44 ± 2.81a	55.74 ± 6.56a
BFM (kg)	10.75 ± 3.82a	11.63 ± 6.75a	9.55 ± 3.70a	9.47 ± 1.88a	14.74 ± 6.17a	5.29 ± 2.16a	25.32 ± 12.80c	15.21 ± 3.77a	28.12 ± 22.80c	9.09 ± 1.92a	18.94 ± 0.06b
TBW (litre)	36.86 ± 2.76a	37.19 ± 3.78a	37.69 ± 2.82a	35.90 ± 0.85a	35.80 ± 1.70a	35.90 ± 2.01a	39.34 ± 3.82a	39.47 ± 2.74a	35.63 ± 5.01a	35.80 ± 1.70a	41.55 ± 2.33a
IF (litre)	20.26 ± 1.51a	20.56 ± 2.04a	20.73 ± 1.45a	19.85 ± 0.35a	21.25 ± 2.33a	19.73 ± 1.11a	21.64 ± 2.07a	21.67 ± 1.43a	19.60 ± 2.74a	19.75 ± 0.92a	22.85 ± 1.34a
EF (litre)	16.58 ± 1.24a	16.58 ± 2.05a	16.96 ± 1.19a	16.20 ± 0.28a	17.35 ± 1.91a	16.17 ± 0.90a	17.73 ± 1.68a	17.71 ± 1.18a	16.03 ± 2.27a	16.15 ± 0.78a	18.70 ± 1.13a
BP (systolic) (mm Hg)	123.64 ± 15.88a	112.82 ± 23.71a	134.63 ± 10.85a	128.00 ± 11.31a	115.00 ± 12.73a	113.14 ± 33.86a	114.60 ± 25.18a	114.29 ± 6.78a	120.00 ± 14.24a	117.50 ± 6.36a	110.50 ± 14.85a
BP (diastolic) (mm Hg)	81.09 ± 19.94a	69.12 ± 16.67a	75.88 ± 10.34a	61.50 ± 16.26a	65.50 ± 2.12a	60.71 ± 15.00a	66.20 ± 15.19a	79.57 ± 7.04a	67.00 ± 11.34a	73.00 ± 1.41a	77.00 ± 24.04a

Values in a row having no letter (a – d) in common are significantly different from each other (p < 0.05).

Analyses of the anthropometric parameters determined in the subjects revealed unsystematic statistically significant differences between the control and the artisans. BMI was higher in drivers, petrol station attendants and welders, but the increase was not statistically significant (p = 0.512). BSA was decreased in spare parts/oil sellers, whereas it increased in the welders (p < 0.05). BFMI was significantly increased in the panel beaters, petrol station attendants, upholsterers and welders (p < 0.05), whereas FFMI was decreased in the upholsterers (p < 0.05). While BFM was increased in panel beaters, upholsterers and welders (p < 0.05), no statistical significant differences were observed between control and the artisans in LBM, TBW, IF and EF.

### Blood pressure

The mean blood pressures of the subjects as depicted in Table 2 indicate that the systolic and diastolic blood pressures of the subjects were in the normotensive range. The auto-electricians however had a borderline high systolic blood pressure (134.63 ± 10.85 mmHg). However, statistical analyses revealed no significant difference in both the systolic and diastolic blood pressures between the control and the artisans (p > 0.05).

### Plasma lipid profiles

The mean values of the investigated blood lipids in the subjects are depicted in Table 3. With a few exceptions, the mean values of these lipids were within the reference ranges prescribed by the American Heart Association [26]. Total cholesterol was slightly higher in the battery chargers while LDL cholesterol was above the reference range in the battery chargers, drivers and petrol station attendants. When the values of the investigated lipids in the artisans were however compared with the controls, certain patterns emerged. With the exception of the painters, the mean plasma concentrations of total cholesterol were significantly higher in the artisans compared with control subjects (p < 0.001). Of the artisans, the battery chargers had the highest plasma total cholesterol (203.50 ± 48.51 mg/dl), followed by the spare parts/oil sellers and drivers respectively. Quantitatively, cholesterol levels of the artisans were between 1.5 and 2.0 times higher than that of the controls. Although there were statistically significant differences in the LDL-cholesterol between control and the artisans, the increases in this parameter exhibited a different pattern compared with total cholesterol. While LDL cholesterol was not significantly different in the painters, upholsterers, spare parts/oil sellers and welders when compared with controls (p > 0.05), values obtained for the auto-mechanics, auto-electricians, drivers, panel beaters, battery chargers and petrol station attendants were significantly higher than controls (p < 0.001). In these groups of artisans, the LDL cholesterol levels were between 1.6 to 2.4 times higher than control subjects. In contrast however, both triglyceride and HDL cholesterol concentrations were not significantly different between control and the artisans (p > 0.05), although the upholsterers tended to have lower HDL cholesterol concentrations when compared with others.

**Table 3:** Blood lead and blood lipid profiles of the subjects. Values are mean ± S.D.

	Subjects										
Parameter	Control	Auto-mechanics	Auto-electricians	Battery chargers	Drivers	Painters (Vehicle)	Panel beaters	Petrol station attendants	Upholsterers	Spare parts/Oil sellers	Welders

Blood lead (μg/dl)	15.78 ± 2.84a	43.98 ± 10.54e	48.90 ± 19.11f	41.13 ± 11.55d	40.99 ± 7.00d	36.10 ± 9.64c	35.99 ± 11.08c	42.53 ± 5.90d	31.61 ± 7.78c	35.79 ± 6.30c	27.00 ± 1.05b
Total chol. (mg/dl)	106.17 ± 16.74a	170.42 ± 43.80c	166.83 ± 43.86b	203.50 ± 48.51e	184.88 ± 64.59d	127.69 ± 19.34a	176.78 ± 52.81c	157.29 ± 27.94b	152.70 ± 61.10b	187.08 ± 57.88d	157.96 ± 87.17b
Triglyceride (mg/dl)	64.17 ± 30.36a	77.29 ± 34.65a	66.90 ± 22.57a	75.29 ± 18.50a	84.19 ± 38.72a	104.85 ± 35.21a	65.47 ± 29.15a	60.58 ± 21.02a	81.61 ± 71.83a	104.50 ± 19.81a	69.68 ± 34.16a
HDL chol. (mg/dl)	50.65 ± 6.96a	45.01 ± 16.11a	51.32 ± 14.04a	40.56 ± 2.55a	39.78 ± 19.80a	49.58 ± 11.54a	41.49 ± 17.21a	40.55 ± 15.42a	27.68 ± 10.43a	52.26 ± 5.37a	73.03 ± 14.17b
LDL chol. (mg/dl)	62.96 ± 24.68a	99.93 ± 33.67b	101.12 ± 42.91b	147.00 ± 49.50c	133.85 ± 81.69b	90.72 ± 35.70a	114.50 ± 30.52b	148.52 ± 29.62c	82.22 ± 59.75a	69.54 ± 3.48a	83.39 ± 44.29a
Total chol./HDL chol.	2.15 ± 0.55a	4.49 ± 3.08b	3.46 ± 1.29a	4.99 ± 0.88b	5.77 ± 4.49b	2.65 ± 0.54a	4.70 ± 2.10b	4.28 ± 1.33a	5.48 ± 0.63b	3.66 ± 1.48a	2.09 ± 0.79a
LDL chol./HDL chol.	1.25 ± 0.47a	2.61 ± 1.49b	2.15 ± 1.18a	3.60 ± 1.00c	4.42 ± 4.26e	1.91 ± 0.82a	3.27 ± 1.71b	4.00 ± 1.11d	3.20 ± 2.03b	1.33 ± 0.07a	1.11 ± 0.39a

Values in a row having no letter (a – f) in common are significantly different from each other (p < 0.05).

The ratios of the lipids, total cholesterol/HDL cholesterol on one hand, and LDL cholesterol/HDL cholesterol on the other hand, are also depicted in Table 3. There were no statistical significant differences between the control and the artisans in total cholesterol/HDL cholesterol ratios (p > 0.05), although there was a tendency towards higher ratios in the artisans. In contrast however, LDL cholesterol/HDL cholesterol ratios were significantly increased in the artisans when compared with the control subjects (p < 0.001). Of the artisans, the highest ratio was observed in the drivers (4.42 ± 4.23), followed closely by the petrol station attendants with 4.00 ± 1.10.

### Blood lead concentrations

The mean blood lead concentrations in the subjects are also depicted in Table 3. Lead levels in the blood of the artisans were significantly higher than that of control (p < 0.001). Of the artisans, the auto-electricians had the highest blood lead level of 48.90 ± 19.11 μg/dl, a value which was 3 times higher than that of control subjects.

### Correlations between blood lead and anthropometric parameters, and blood lead and investigated lipids

There was no significant correlation between blood lead levels and any of the anthropometric parameters (p > 0.05) (Data not shown). However, a positive significant correlation was observed between blood lead and total cholesterol (r = 0.372; p = 3.0 × 10^-5^) and blood lead and LDL cholesterol (r = 0.283; p = 0.001). We also analysed our data to see whether any correlation existed among the anthropometric parameters on one hand and anthropometric parameters and investigated lipids on the other hand. The following were observed:

1. A significant positive correlation between age and systolic blood pressure (r = 0.325; p = 3.0 × 10^-4^).

2. A significant positive correlation between age and diastolic blood pressure (r = 0.232; p = 0.007).

3. A significant positive correlation between age and BMI (r = 0.449; p = 1 × 10^-5^).

4. A significant positive correlation between weight and triglyceride (r = 0.275; p = 0.006).

5. A significant positive correlation between age and weight (r = 0.418; p = 5 × 10^-5^).

6. A significant positive correlation between BMI and triglyceride (r = 0.354; p = 6 × 10^-4^).

7. A significant positive correlation between BSA and LDL cholesterol (r = 0.202; p = 0.035).

8. A significant positive correlation between age and BSA (r = 0.390; p = 1 × 10^-4^).

9. A significant positive correlation between BMI and systolic blood pressure (r = 0.371; p = 7 × 10^-4^).

10. A significant positive correlation between BMI and diastolic blood pressure (r = 0.424; p = 1 × 10^-4^).

## Discussion

During the past decade, a vast amount of evidence has confirmed that lipid and lipoprotein abnormalities play a major role in the pathogenesis and progression of atherosclerosis and cardiovascular diseases [23-27]. These chronic degenerative disorders have become a growing health problem worldwide.

In African populations, dyslipidemia as a risk factor for cardiovascular disease and increasing incidents of death due to cardiovascular disease in both urbanised and underdeveloped rural countries have been reported [34]. There is also increasing evidence that environmental factors contribute to this dyslipidemia [35]. In this present study, we evaluated the distribution of some blood lipids in a population of artisans who are occupationally exposed to lead, an occupational and environmental pollutant. We found that majority of the artisans had HDL cholesterol and triglyceride levels not significantly different from controls. On the contrary, total cholesterol levels in the artisans were between 1.5 and 2.0 times higher than controls. In addition, LDL cholesterol in some of the artisans was considerably higher when compared with controls. To our knowledge, the distribution of blood lipids in artisans in Nigeria has not been reported in the literature. We were compelled to compare our data with the guidelines of risk factors for cardiovascular disease given by the American Heart Association [26]. According to these guidelines, blood pressure < 130/85 mmHg; total cholesterol < 200 mg/dl; triglycerides < 200 mg/dl; HDL > 40 mg/dl and LDL < 130 mg/dl, are favourable risk factors. In addition, certain lipid ratios like total cholesterol/HDL cholesterol and the LDL cholesterol/HDL cholesterol ratio also correlate with cardiovascular disease. The recommended ratios for the two are ≤ 3.5 [26]. Indications from this comparison are that while the HDL cholesterol and triglyceride concentrations of both the controls and artisans were within the acceptable range prescribed by the American Heart Association, the battery chargers with total cholesterol of 203.50 ± 48.51 mg/dl and LDL cholesterol of 147.00 ± 49.50 mg/dl, drivers with LDL cholesterol of 133.85 ± 81.69 mg/dl, and petrol station attendants with LDL cholesterol of 148.52 ± 29.62 mg/dl, seem to have unfavourable risk profiles for cardiovascular disease when compared with other artisans and control. Furthermore, the LDL/HDL ratio of the artisans indicate that battery chargers, petrol station attendants and drivers seem to have a greater risk of cardiovascular disease when compared with other artisans and controls. As regards total cholesterol/HDL cholesterol ratio, the auto-mechanics, battery chargers, drivers, panel beaters, petrol station attendants, spare parts/oil sellers and upholsterers, seem to be at risk of cardiovascular disease. Although cholesterol levels are lower in African population when compared with their American counterparts [36,37], the findings of the present investigation indicate that exposure to lead alters the metabolism of cholesterol and thus increases the risk of cardiovascular disease and atherosclerosis in lead-exposed subjects. Although correlations do not imply causality, the observation of a positive relationship between lead and total cholesterol on one hand, and lead and LDL cholesterol on the other hand, seems to support these experimental findings.

HDL and LDL are two of the four main groups of plasma lipoproteins that are involved in lipid metabolism and the exchange of cholesterol, cholesterol ester and triglycerides between tissues [38-40]. Numerous population studies have shown an inverse correlation between plasma HDL levels and risk of cardiovascular disease, implying that factors associated with HDL protect against atherosclerosis. Some of these factors appear to have anti-oxidant and anti-inflammatory effects which may obviate processes that initiate atherogenesis [41,42]. Epidemiological studies have also shown that elevated concentrations of total or LDL cholesterol in the blood are powerful risk factors for coronary disease [43]. Most extra-hepatic tissues, although having a requirement for cholesterol, have low activity of the cholesterol biosynthetic pathway. Their cholesterol requirements are supplied by LDL, which is internalised by receptor-mediated endocytosis. A major function of HDL cholesterol is to enhance reverse cholesterol transport by scavenging excess cholesterol from peripheral tissues followed by esterification through lecithin:cholesterol acyltransferase and delivering it to the liver and steroidogenic organs for subsequent synthesis of bile acids and lipoproteins and eventual elimination from the body [44,45]. This role of HDL has been shown to be responsible for its atheroprotective properties. HDL cholesterol also regulates the exchange of proteins and lipids between various lipoproteins [46]. In addition, HDL provides the protein components required to activate lipoprotein lipase which releases fatty acids that can be oxidised by the β-oxidation pathway to release energy [38-40]. Most importantly, HDL can inhibit oxidation of LDL as well as the atherogenic effects of oxidised LDL by virtue of its antioxidant property [45]. The observation of increased total plasma cholesterol and LDL cholesterol levels, and normal HDL cholesterol levels in the artisans in this study suggests that reverse cholesterol transport in these artisans was not affected by lead exposure, rather cholesterol synthesis and transport to the peripheral tissues might be affected. It is possible that lead increases the activity of 3-hydroxy-3-methylglutaryl coenzyme A (HMG CoA) reductase (the rate-limiting enzyme in cholesterol biosynthesis) and reduces the number/affinity of LDL receptors for cholesterol. Further studies that we are pursuing in this laboratory are addressing this hypothesis.

Exposure to lead has been shown to be a significant risk factor for the development of hypertension [47,48] and this is supported by experimental animal studies in several species as well as epidemiological studies of blood pressure in relation to blood lead levels [47-49]. Most, but not all, of the studies have demonstrated that blood lead increases from 10 to 25 μg/dl are associated with systolic and diastolic blood pressure increases of 1.4–8 mmHg and 1.2–4 mmHg, respectively [48]. Mean blood lead levels observed in the artisans in this study were between 2.5 and 3.0 times higher than control subjects. Even though job experience indicated that lead exposure has been going on in these artisans for 4 to 26 years, only the auto-electricians had a borderline high systolic blood pressure as prescribed by the American Heart Association. It remains to be established in these artisans the threshold value of blood lead that might be associated with increased blood pressure.

In addition to blood lipids, other factors such as age, physical activity, genetics, body composition, alcohol intake, tobacco use and body fat distribution, contribute significantly to risk of cardiovascular disease [26,50]. All the subjects who participated in this study were non-smokers and all consume cassava-based meal as their carbohydrate source. Alcohol intake was limited to only six of the auto-mechanics who consume 1 bottle of beer per day. By virtue of the demands of their occupations, most of the artisans are physically active and this might account for the normal levels of HDL cholesterol observed in them [35]. The anthropometric parameters measured in the artisans did not reveal any statistically significant difference between the control subjects and artisans, thus suggesting that these factors may not play any significant role in the dyslipidemia observed in these artisans.

The increased plasma cholesterol levels observed in these artisans raises a few questions. Since erythrocyte cholesterol reflects plasma cholesterol, it might be interesting to investigate cholesterol levels in the erythrocytes in lead exposure and how this affects cholesterol:phospholipid ratio in both plasma and erythrocytes. The consequences of these alterations might probably explain alterations observed in cation fluxes in the erythrocyte membrane in lead exposure. Studies going on at present in this laboratory are directed towards addressing these questions.
